# Low Serum Bicarbonate Predicts Residual Renal Function Loss in Peritoneal Dialysis Patients

**DOI:** 10.1097/MD.0000000000001276

**Published:** 2015-08-07

**Authors:** Tae Ik Chang, Ea Wha Kang, Hyung Woo Kim, Geun Woo Ryu, Cheol Ho Park, Jung Tak Park, Tae-Hyun Yoo, Sug Kyun Shin, Shin-Wook Kang, Kyu Hun Choi, Dae Suk Han, Seung Hyeok Han

**Affiliations:** From the Department of Internal Medicine (TIC, EWK, SKS), NHIS Medical Center, Ilsan Hospital, Goyangshi, Gyeonggi-do; Department of Internal Medicine (HWK, GWR, CHP, JTP, T-HY, S-WK, KHC, DSH, SHH), College of Medicine, Yonsei University, Seoul; and Brain Korea 21 for Medical Science (S-WK), Severance Biomedical Science Institute, Yonsei University, Seoul, Republic of Korea.

## Abstract

Low residual renal function (RRF) and serum bicarbonate are associated with adverse outcomes in peritoneal dialysis (PD) patients. However, a relationship between the 2 has not yet been determined in these patients. Therefore, this study aimed to investigate whether low serum bicarbonate has a deteriorating effect on RRF in PD patients.

This prospective observational study included a total of 405 incident patients who started PD between January 2000 and December 2005. We determined risk factors for complete loss of RRF using competing risk methods and evaluated the effects of time-averaged serum bicarbonate (TA-Bic) on the decline of RRF over the first 3 years of dialysis treatment using generalized linear mixed models.

During the first 3 years of dialysis, 95 (23.5%) patients became anuric. The mean time until patients became anuric was 20.8 ± 9.0 months. After adjusting for multiple potentially confounding covariates, an increase in TA-Bic level was associated with a significantly decreased risk of loss of RRF (hazard ratio per 1 mEq/L increase, 0.84; 0.75–0.93; *P* = 0.002), and in comparison to TA-Bic ≥ 24 mEq/L, TA-Bic < 24 mEq/L conferred a 2.62-fold higher risk of becoming anuric. Furthermore, the rate of RRF decline estimated by generalized linear mixed models was significantly greater in patients with TA-Bic < 24 mEq/L compared with those with TA-Bic ≥ 24 mEq/L (−0.16 vs −0.11 mL/min/mo/1.73 m^2^, *P* < 0.001).

In this study, a clear association was found between low serum bicarbonate and loss of RRF in PD patients. Nevertheless, whether correction of metabolic acidosis for this indication provides additional protection for preserving RRF in these patients is unknown. Future interventional studies should more appropriately address this question.

## INTRODUCTION

Decline of residual renal function (RRF) is an independent risk factor for adverse outcomes in patients with chronic kidney disease (CKD), and remains important even after dialysis has been started.^1–3^ It is associated with fluid overload,^4^ anemia,^5^ inflammation,^6^ and malnutrition,^7^ and is also a strong predictor of mortality in these patients.^8–10^ Thus, preserving RRF is now considered to be one of the primary goals in managing patients on dialysis.

Recently, metabolic acidosis, which usually manifests as low serum bicarbonate, has emerged as a modifiable factor that is strongly associated with increased mortality in CKD patients.^11–16^ Correction of acidosis confers kidney protection and also slows glomerular filtration rate (GFR) decline in nondialysis-dependent CKD patients.^17–24^ This considerable evidence has raised the possibility that low serum bicarbonate levels may be associated with rapid decline or complete loss of RRF, and can thereby contribute to poor outcomes in peritoneal dialysis (PD) patients. Nevertheless, there are only a few studies that have investigated whether low serum bicarbonate predicts, or has a deteriorating effect on RRF in these patients. Therefore, the purpose of this study was to determine the associations between low serum bicarbonate and loss of RRF in a large prospective cohort of incident patients undergoing PD.

## METHODS

### Patients

**FIGURE 1:**
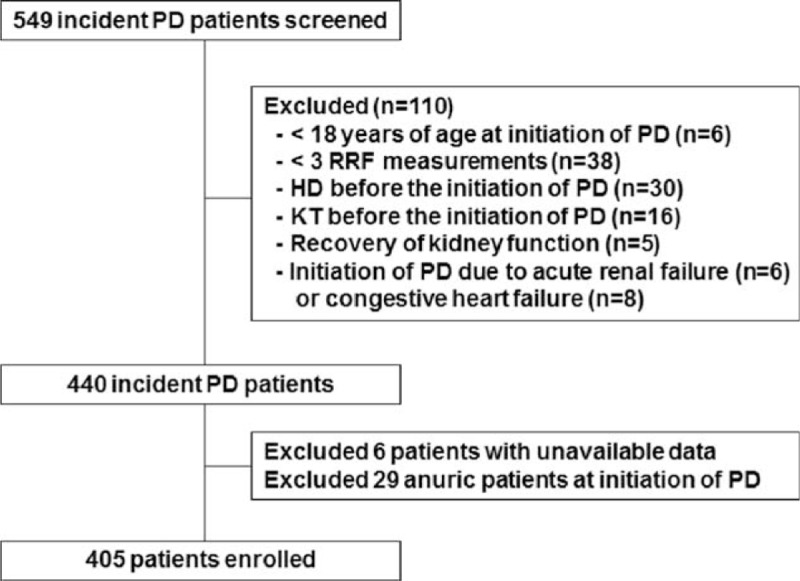
Flow chart of participants in the cohort. HD = hemodialysis, KT = kidney transplant, PD = peritoneal dialysis, RRF = residual renal function.

The study population included 549 end-stage renal disease (ESRD) patients who started PD at Yonsei University Severance Hospital or NHIS Ilsan Hospital between January 2000 and December 2005. Exclusion criteria were <18 years of age at initiation of PD, <3 RRF measurements, prior history of hemodialysis (HD) or a kidney transplant before PD was initiated, recovery of kidney function, or initiation of PD for other reasons such as acute renal failure or congestive heart failure. Because we were interested in the decline of RRF, patients were also excluded when they were already anuric at dialysis initiation. Therefore, this prospective observational study included a total of 405 incident patients (Figure 1). The study was carried out in accordance with the Declaration of Helsinki and approved by the Institutional Review Board of Ilsan Hospital Clinical Trial Center. We obtained informed written consent from all participants involved in our study.

### Data Collection

Demographic and clinical data were collected at the beginning of PD. These included age, sex, body mass index (BMI) calculated as weight/(height),^2^ cause of ESRD, presence of diabetes, and medications. Charlson Comorbidity Index was scored at the start of PD which was a scoring system that includes weighing factors on important concomitant diseases.^25^

Data on RRF were collected within 3 months of PD initiation and followed by 6-month intervals thereafter. RRF was expressed as residual GFR, calculated as the average urea, and creatinine clearance from a 24-hour urine collection.^26^ Residual GFR was considered null when urine output was <200 mL/d. When a patient reached a residual GFR below this level at 2 successive time points, we defined the patient as anuric from the first time point of the 2 measurements.

Laboratory and dialysis characteristics obtained at the time of initial dialysis adequacy measurement were considered as baseline values, and included serum bicarbonate concentrations, blood urea nitrogen, serum creatinine, total cholesterol, calcium, phosphorus, serum albumin, serum C-reactive protein (CRP) levels, Kt/V urea, dialysis volume, PD ultrafiltration, normalized protein catabolic rate (nPCR), percentage of lean body mass (%LBM), and types of dialysate. Serum total CO_2_, which is generally used as an indirect measure of serum bicarbonate concentration,^27^ was measured by an electrode-based method (UniCel DXC 800; Beckman Coulter, Inc, CA) and recorded longitudinally throughout the follow-up period. Time-averaged serum bicarbonate (TA-Bic) was calculated as an average of the mean of bicarbonate measurements every 3 months.

### Statistical Analysis

All values are expressed as the mean ± standard deviation or percentages. Statistical analyses were performed using SAS version 9.2.3 (SAS Institute Inc, Cary, NC) and the software package R version 3.0.2. Comparisons were made by Student *t* test for continuous variables and by the χ^2^ test for categorical variables as required. The Kolmogorov–Smirnov test was used to determine the normality of the distribution of parameters. If data did not show a normal distribution, they were expressed as the median and interquartile range (or after log-transformation), and were compared using the Mann–Whitney *U* test or Kruskal–Wallis test. For comparisons, based on the cutoff values used in our laboratory, patients were further divided into 3 groups: <24 mEq/L (low), 24 to 30 mEq/L (normal), and >30 mEq/L (high) of TA-Bic levels.

Time to becoming anuric during the first 3 years of dialysis therapy was estimated and compared between groups using the cumulative incidence competing risk method and the K-sample test developed by Gray.^28^ Data for switching to HD, kidney transplantation, loss to follow-up, and death were censored in the analysis. To determine risk factors for becoming anuric, competing risks multivariate Cox regression was performed, as described by Fine and Gray,^29^ and 3 different models were constructed; adjustments in model 1: demographic and clinical parameters, including age, sex, BMI, Charlson Comorbidity Index score, sevelamer hydrochloride treatment, antihypertensive medications, and peritonitis rate; model 2: model 1 plus dialysis-specific parameters, including types of PD solutions (type of buffers, final pH of solutions, and use of icodextrin), prescribed dialysate volume, PD ultrafiltration, total Kt/V urea, nPCR, and %LBM; model 3: model 2 plus laboratory parameters, including serum hemoglobin, serum albumin, total cholesterol, calcium, phosphorus, serum ferritin, and CRP. For all survival analyses, the Cox model proportionality assumption was confirmed by testing Schoenfeld residuals. First-order interaction terms between covariates were examined for all models, but there was no evidence of an interaction between those covariates. The results are expressed as a hazard ratio (HR) and 95% confidence interval (CI). We further modified the above Cox regression analyses by using a restricted cubic spline model with 2 degrees of freedom to illustrate systemic relations between serum bicarbonate levels and the risk of anuric event. This method generally examines nonlinear associations to avoid issues caused by potential inappropriate assumptions concerning linearity.^30^

In addition, generalized linear mixed models for repeated measures were applied to analyze the effects of TA-Bic on the decline in RRF over the first 3 years of dialysis treatment. The slope of the decline in RRF over time was also calculated and compared by a linear mixed model, and was expressed as the estimate coefficient and 95% CI (mL/min/mo/1.73 m^2^). Furthermore, an additional comparison was made after adjusting for all repeatedly measured covariates included in multivariate Cox regression analyses. A *P* value <0.05 was considered statistically significant.

## RESULTS

### Patient Characteristics

From the total of 549 patients, we excluded 144 patients, thus 405 patients were included in the final analysis (Figure 1). Compared with the included patients, the excluded patients were significantly older and more frequently had a higher comorbidity score. Other baseline characteristics were not different between included and excluded patients. Table 1 shows the baseline characteristics of the 405 included patients. The mean age of the patients was 59.4 years (range, 22–85 years), 53.8% were males, and 52.1% were diabetic. The median TA-Bic level was 26.0 mEq/L (range, 16.6–33.9 mEq/L). All baseline characteristics were not different among patients with TA-Bic < 24 mEq/L, 24 to 30 mEq/L, and >30 mEq/L, with the exception of types of PD solutions; the proportion of patients who were either on low (35 mEq/L) or high (40 mEqL) lactate-buffered solutions and were on low pH (≤5.5) solutions was significantly higher in the lower TA-Bic group compared with the higher TA-Bic group. Conversely, patients in the higher TA-Bic group were more likely to use bicarbonate-/lactate-buffered solutions (25 and 15 mEq/L, respectively) and high pH (>5.5) solutions.

**TABLE 1:**
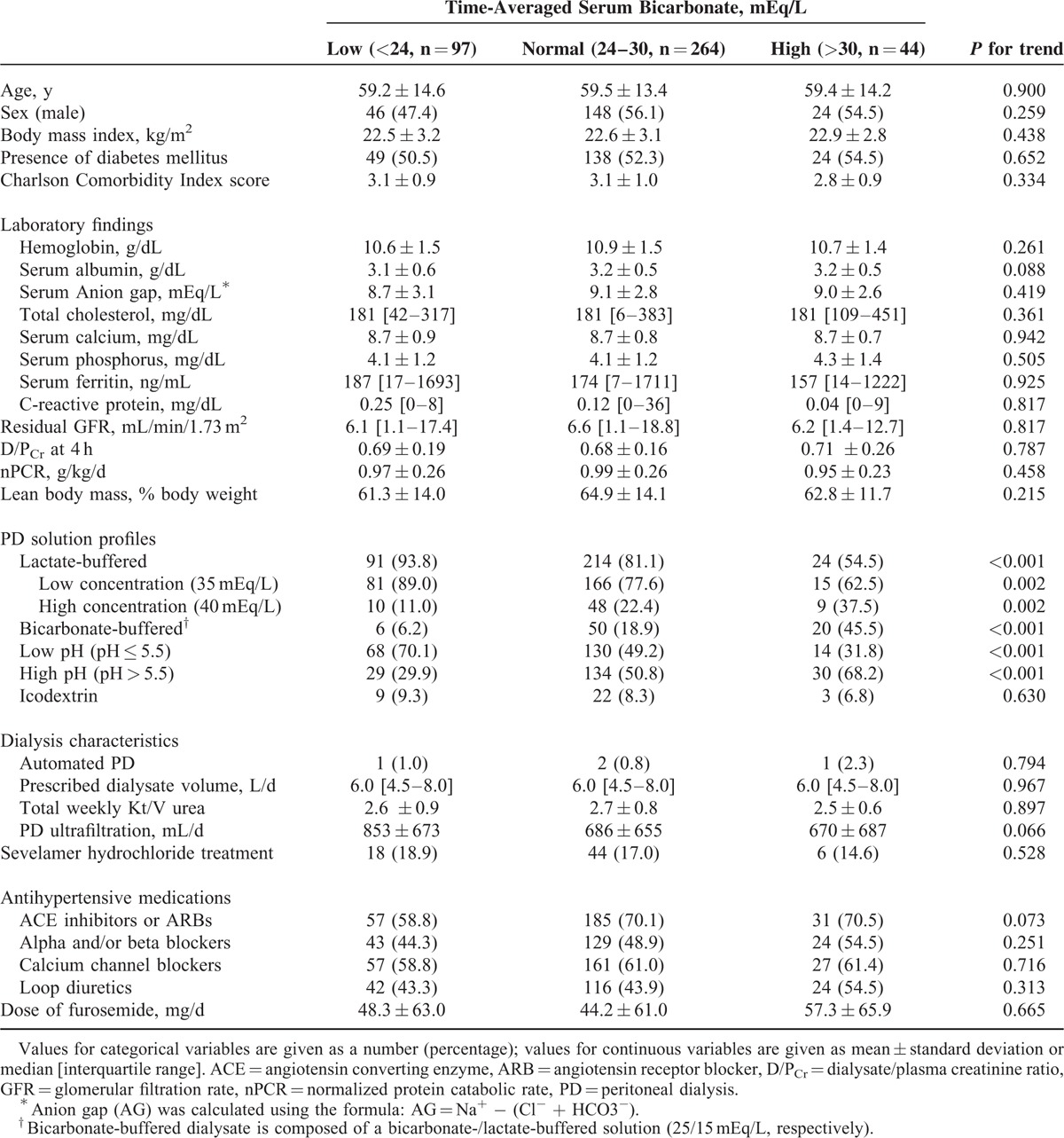
Baseline Characteristics of Study Subjects Stratified by Time-Averaged Serum Bicarbonate

Patients were followed for a mean duration of 25.8 months. During the follow-up period, a total of 299 episodes of peritonitis were observed in 171 patients (total 871.3 patient-years) and overall peritonitis rate was 0.34 per patient-year. Mean peritonitis rates were 0.29 (64 episodes per 221.9 patient-years), 0.37 (203 episodes per 552.9 patient-years), and 0.33 (32 episodes per 96.4 patient-years) in low, normal, and high TA-Bic groups, respectively (*P* for trend = 0.126). In addition, serum albumin concentration, which is usually recognized as a nutritional and inflammatory marker, did not differ between the groups during follow-up (3.3 ± 0.7, 3.2 ± 0.6, and 3.2 ± 0.6 g/dL in the 3 groups of TA-Bic: <24, 24–30, and >30 mEq/L, respectively; *P* = 0.859 by comparison of difference in overall change by linear mixed models with repeated measures).

### Loss of RRF

**FIGURE 2:**
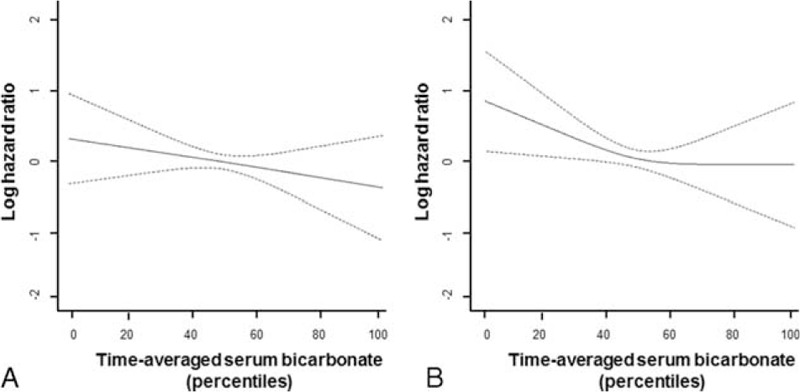
Cubic spline regression models of the log-hazard (with 95% confidence interval and 2 degrees of freedom) of anuric event according to the percentile of time-averaged serum bicarbonate levels. (A) Univariate model and (B) multivariate model adjusted for age, sex, body mass index, Charlson Comorbidity Index score, medications (sevelamer, antihypertensive), peritonitis rate, types of peritoneal dialysis solutions (lactate/bicarbonate buffered solution, final pH of solution, use of icodextrin), dialysate volume, peritoneal dialysis ultrafiltration, total Kt/V urea, normalized protein catabolic rate, percentage of lean body mass, serum hemoglobin, serum albumin, total cholesterol, calcium, phosphorus, serum ferritin, and C-reactive protein levels.

RRF was measured at least 4 times in 287 (70.9%), 5 times in 242 (59.8%), 6 times in 209 (51.6%), and 7 times in 166 (41.0%) patients. During the first 3 years of dialysis, 95 (23.5%) patients became anuric. The mean time until patients became anuric was 20.8 ± 9.0 months. The results of the cubic splines graph illustrating the association between TA-Bic levels (in percentiles) and the risk of anuric event are shown in Figure 2. A trend toward increased risk of anuria was observed in PD patients with lower TA-Bic levels. The lowest quintile of serum TA-Bic appeared to be associated with the greatest risk of becoming anuria.

**FIGURE 3:**
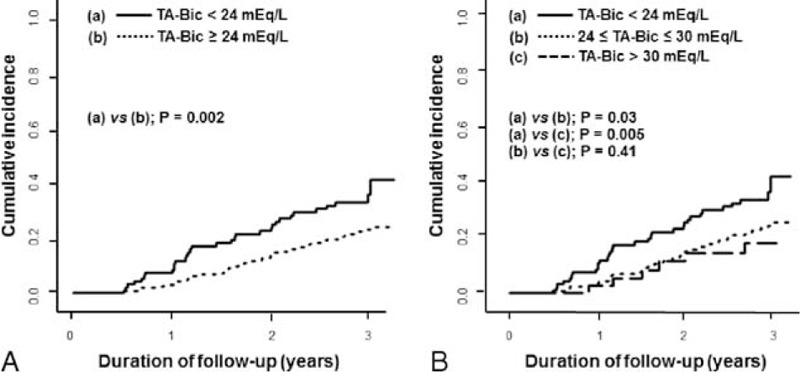
The cumulative incidence curves for the time until complete loss of residual renal function between groups based on the level of 24 mEq/L (A) and the level of <24, 24 to 30, and >30 mEq/L (B) of TA-Bic levels. TA-Bic = time-averaged serum bicarbonate.

In addition, the cumulative incidence of complete RRF loss was significantly lower in patients with higher TA-Bic level compared with patients with TA-Bic level <24 mEq/L (Figure 3). The HRs for full loss of RRF within 3 years after dialysis initiation are shown in Table 2. Models with various adjustment showed that the association between the 2 was significant and consistent. Using serum TA-Bic as a continuous variable, an HR for total loss of RRF was 0.84 per 1 mEq/L higher for TA-Bic (95% CI, 0.75–0.93; *P* = 0.002), indicating that higher TA-Bic was significantly associated with decreased risk of complete RRF loss. Moreover, patients with a TA-Bic < 24 mEq/L conferred a 2.62-fold higher risk of becoming anuric compared with patients with a TA-Bic ≥ 24 mEq/L.

**TABLE 2:**
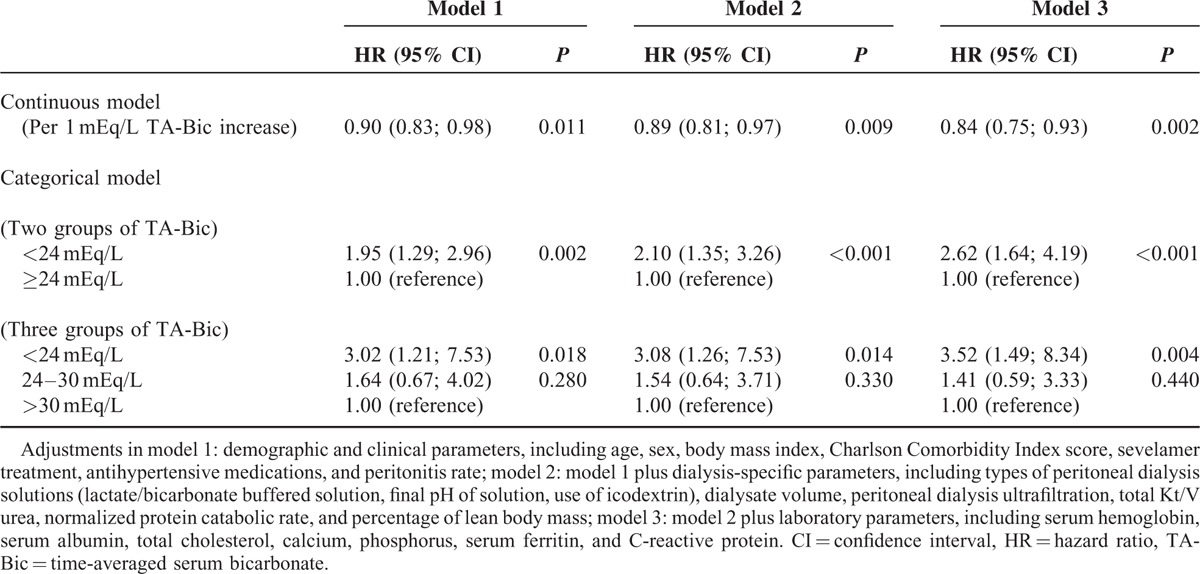
Multivariable Cox Regression Analyses for the Risk of Complete Loss of Residual Renal Function

### Decline of RRF

**FIGURE 4:**
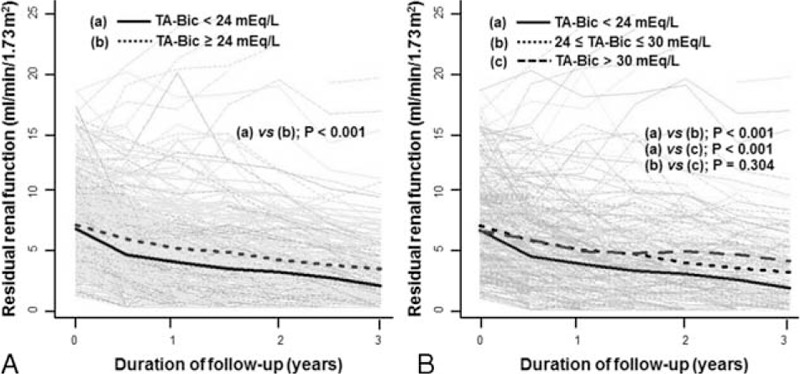
Changes in residual renal function over time between groups based on the level of 24 mEq/L (A) and the level of <24, 24 to 30, and >30 mEq/L (B) of TA-Bic levels. Gray lines represent individual patient measurements, and solid and dash lines represent predicted slopes. TA-Bic = time-averaged serum bicarbonate.

We evaluated the effects of TA-Bic on the decline of RRF over time using generalized linear mixed models for repeated measures (Figure 4). The overall rates of RRF decline after the start of PD were significantly greater in patients with TA-Bic < 24 mEq/L compared with those with TA-Bic ≥ 24 mEq/L (−0.15 vs −0.11 mL/min/mo/1.73 m^2^, *P* < 0.001). When patients were divided into 3 groups of TA-Bic (<24, 24–30, and >30 mEq/L), the monthly change in RRF decline was significantly higher in patients in the low TA-Bic group (−0.15 mL/min/mo/1.73 m^2^) compared with those in the normal (−0.11 mL/min/mo/1.73 m^2^, *P* < 0.001) and the high TA-Bic (−0.09 mL/min/mo/1.73 m^2^, *P* < 0.001) groups, respectively. When the curvature of GFR decline was alternatively modeled by analyzing log-transformed data over the entire study period, similar results were found (data not shown), indicating that a lower TA-Bic level was associated with the rapid decline in RRF within 3 years after dialysis was initiated.

Even after additional interaction terms for time with potential confounders were included in the Cox regression analyses, this association between the 2 remained significant and consistent (Table 3). Furthermore, to verify whether the results were influenced by baseline RRF, we repeated all mixed model analyses in subgroups of patients with a high or a low baseline RRF based on the median residual GFR level (6.41 mL/min/1.73 m^2^). In this additional analysis, the observed effects in the different subgroups were similar to those in the crude analyses, and this did not alter our conclusions (data not shown).

**TABLE 3:**
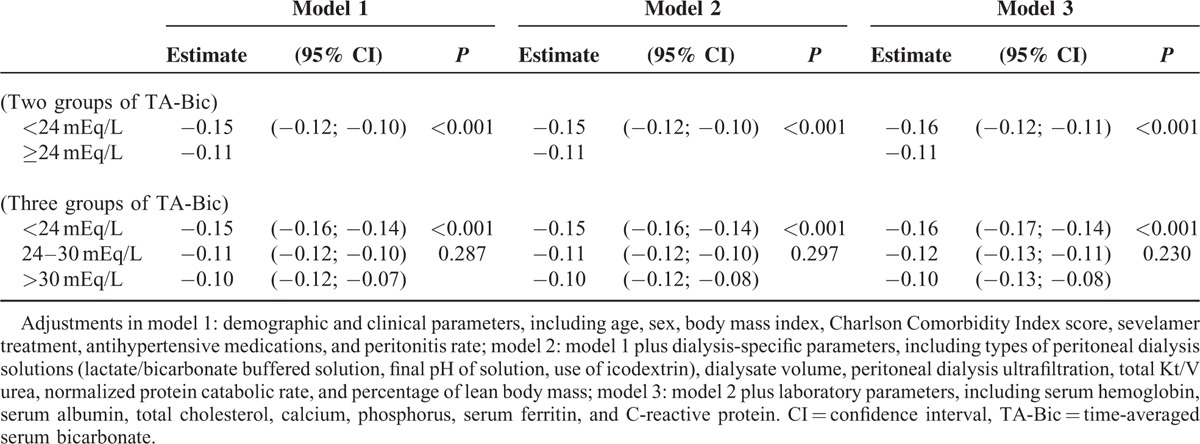
Multivariate Adjusted Linear Mixed Models for Monthly Change in Residual Renal Function (mL/min/mo/1.73 m^2^)

## DISCUSSION

In this study, we sought to delineate the relationship between serum bicarbonate levels and RRF in our ESRD cohort of patients with PD. We showed that a low TA-Bic level independently predicted complete loss of RRF and was also significantly associated with a rapid decline in RRF. This finding suggests that metabolic acidosis exerts a detrimental effect on RRF decline. Thus, correcting low bicarbonate level can be considered as a therapeutic option to preserve RRF even after dialysis initiation.

Although ESRD patients are treated with dialysis, previous studies including ours have clearly showed that metabolic acidosis is common and significantly associated with adverse outcomes in these patients.^11–16,31–34^ In fact, metabolic acidosis is reported to be an important cause of many deleterious metabolic consequences, including protein-energy wasting, inflammation, bone disease, and disturbance in endocrine function.^31–34^ Furthermore, there is evidence that indicates there is a significant association between low serum bicarbonate levels and increased mortality in these patients.^11–16^ In addition to these unfavorable effects, metabolic acidosis has also been highly related to kidney damage and rapid decline of GFR in patients with CKD before dialysis.^35,36^ These findings were further supported by several recent clinical trials showing that treatment of metabolic acidosis with oral alkali or base-producing fruits and vegetables decreased markers of renal injury and attenuated progression of kidney disease in these patients.^17–24^ Of note, the importance of preserving RRF is well established in patients with ESRD, and decline of RRF is a critical prognostic factor in these patients.^1–10^ Moreover, we have recently shown that a low TA-Bic is associated with increased mortality, and the TA-Bic level also positively correlated with RRF in the same cohort of incident PD patients.^16^ These findings suggest a possible causal link between acidosis-induced hastened decline in RRF and adverse outcomes. Further investigations are required to establish the direct relationship between low serum bicarbonate and loss of RRF in these patients, which can provide a rationale for additional therapeutic strategy to improve clinical outcomes.

Our findings deserve attention as we clarified the association between acidosis and RRF decline using different statistical methods with rigorous adjustment. In this study, we clearly showed that low serum bicarbonate level was an independent predictor of full loss of RRF after dialysis initiation in the multivariate Cox proportional hazards model which adjusted for potentially multiple confounding covariates, including demographic, clinical, laboratory, and dialysis-specific data. In addition, the rate of RRF decline estimated by generalized linear mixed models for repeated measures was significantly greater in patients with TA-Bic < 24 mEq/L compared with those with TA-Bic ≥ 24 mEq/L. Increased RRF decline in patients with low bicarbonate concentrations was also confirmed when we compared the rate of RRF decline in the 3 different groups of TA-Bic levels (<24, 24–30, and >30 mEq/L) using the same analytical method. In this regard, our robust finding highlights the importance of acidosis as a negative effector on RRF in PD patients even after dialysis initiation.

The underlying mechanisms for rapid RRF decline in CKD patients with lower serum bicarbonate levels are unclear. However, there is compelling evidence that kidney injury is attributed to increased ammoniogenesis in the proximal tubule and increased production of inflammatory mediators such as endothelin, aldosterone, and angiotensin II caused by acid load.^37–39^ In particular, animal studies using a 5/6 nephrectomy model, which is the classic experimental model for human CKD, showed that increased ammoniogenesis-mediated tubulointerstitial injury through activation of the compliment cascade^37^ and metabolic acidosis induced decline in GFR through endothelin A receptors.^38^ In addition, acid retention associated with reduced GFR augments kidney injury through increased kidney levels of endothelin-1, aldosterone, and angiotensin II in the 2/3 nephrectomy model.^39^ Interestingly, all these studies showed that alkali supplements ameliorated kidney injury, further supporting the detrimental role of acidosis in kidney function deterioration. With such experimental-based background in mind, 6 randomized controlled trials have been conducted to date and most of the studies showed positive results that favor correcting acidosis in terms of preserving kidney function.^19–24^ However, all study subjects were in CKD stage 1 to 4 before dialysis, thus it is unknown whether such beneficial effects of correcting acidosis may persist in patients receiving dialysis. Notably, one previous study in a Chinese PD population reported that continuous ambulatory PD patients with normal anion gap metabolic acidosis had better RRF than those with increased anion gap metabolic acidosis and those without acidosis. The authors presumed that increased urinary excretion of bicarbonate in patients with better RRF may be responsible for the occurrence of metabolic acidosis.^40^ However, their findings have not been validated by other studies and should be interpreted with caution. In fact, this study was limited by relatively small sample size (20 continuous ambulatory PD patients), thus resulting in insufficient statistical power. In addition, they did not provide multivariable-adjusted analysis data. Moreover, their cross-sectional study captured only one measurement of each parameter at a specific time point, thus could not represent the whole dialysis period. In contrast, we presented TA-Bic levels and residual GFR using longitudinal follow-up data, and provided convincing association between bicarbonate level and RRF with rigorous adjustment. In this regard, our findings call for well-designed randomized controlled trials with alkali therapy or its alternatives to quantify the causal effect of low serum bicarbonate on reduced GFR and to provide complimentary or adjunctive therapies for preserving RRF in patients with ESRD on dialysis.

Optimal bicarbonate level has not yet been determined in PD patients. Our prior work showed that mortality risk was the lowest in PD patients with TA-Bic ≥ 24 mEq/L.^16^ In line with this finding, the present study also showed that decline in residual GFR was slower in those with bicarbonate levels ≥24 mEq/L. The threshold level based on our studies is slightly higher than the bicarbonate level recommended by the National Kidney Foundation Disease Outcomes Quality Initiative.^41^ However, the suggested cutoff value was merely based on the effects of acidosis on metabolic bone disease and nutritional parameters.^41^ Interestingly, post hoc analysis of the African American Study of Kidney Disease and Hypertension (AASK) trial showed that higher levels of serum bicarbonate (≥25 mmol/L) within the normal range were associated with better survival and kidney outcomes. Moreover, the guidelines from the United Kingdom Renal Association suggest a higher threshold of bicarbonate in PD patients than in HD patients.^42,43^ Thus, future works should address this issue on whether PD patients will benefit from higher target of bicarbonate level.

There were several limitations to this present study. As aforementioned, this is an observational study with a relatively small sample size. Hence, the causality of our findings needs further confirmation. In fact, metabolic acidosis can be merely a secondary phenomenon as kidney function declines because it generally occurs due to a loss of buffer capacity by the kidney. However, acidotic milieu itself can cause tubular injury and activate the inflammatory process, thus it is very likely that kidney failure and acidosis work together in a vicious cycle and ultimately result in exacerbation of kidney injury. In addition, considering that the mechanism contributing to RRF decline is multifactorial,^1–3^ lack of information to further explain loss of RRF in these patients is another drawback. Data representing overall volume status (body weight changes, dietary water intake, and a bioelectrical impedance analysis) and nutritional status (subjective global assessment, anthropometry, and dietary protein intake) were not available for analysis. Other information related to worsening RRF, such as use of nephrotoxic agents or history of infection, was not included. Despite these limitations, this study showed that a low TA-Bic level independently predicted full loss of RRF during the first 3 years of dialysis, and was significantly associated with rapid decline of RRF in PD patients, even after extensive adjustment for demographic, clinical, laboratory, and dialysis-specific covariates.

In conclusion, this study showed a clear association between low serum bicarbonate and loss of RRF in PD patients. However, it has not been determined whether correction of metabolic acidosis for this indication provides additional protection for preserving RRF in these patients. Therefore, future interventional studies should more appropriately address this question.
